# Identification and characterization of *petiolule-like pulvinus* mutants with abolished nyctinastic leaf movement in the model legume *Medicago truncatula*

**DOI:** 10.1111/j.1469-8137.2012.04268.x

**Published:** 2012-08-14

**Authors:** Chuanen Zhou, Lu Han, Chunxiang Fu, Maofeng Chai, Wenzheng Zhang, Guifen Li, Yuhong Tang, Zeng-Yu Wang

**Affiliations:** 1 Forage Improvement Division, The Samuel Roberts Noble Foundation2510 Sam Noble Parkway, Ardmore, OK, 73401, USA; 2 Plant Biology Division, The Samuel Roberts Noble Foundation2510 Sam Noble Parkway, Ardmore, OK, 73401, USA

**Keywords:** leaf movement, *Medicago truncatula*, nyctinasty, *PETIOLULE-LIKE PULVINUS*, pulvinus

## Abstract

- Leaves of many plant species open during the day and fold at night. Diurnal leaf movement, named nyctinasty, has been of great interest to researchers since Darwin's time. Nyctinastic leaf movement is generated by the pulvinus, which is a specialized motor organ located at the base of leaf and leaflet. The molecular basis and functional reason behind nyctinasty are unknown.
- In a forward screening of a retrotransposon-tagged mutant population of *Medicago truncatula*, four *petiolule-like pulvinus* (*plp*) mutant lines with defects in leaf movement were identified and characterized.
- Loss of function of *PLP* results in the change of pulvini to petiolules. *PLP* is specifically expressed in the pulvinus, as demonstrated by quantitative reverse-transcription polymerase chain reaction analysis, expression analysis of a *PLP* promoter-β-glucuronidase construct in transgenic plants and *in situ* hybridization. Microarray analysis revealed that the expression levels of many genes were altered in the mutant during the day and at night. Crosses between the *plp* mutant and several leaf pattern mutants showed that the developmental mechanisms of pulvini and leaf patterns are likely independent.
- Our results demonstrated that *PLP* plays a crucial role in the determination of pulvinus development. Leaf movement generated by pulvini may have an impact on plant vegetative growth.

## Introduction

The metabolism, physiology and behavior of plants are orchestrated by diurnal rhythms generated by the rising and setting of the sun (McClung, 2006; de Montaigu *et al*., 2010). The ability to anticipate such circadian rhythms gives plants fitness advantages, such as enhanced chlorophyll content and improved photosynthesis (Dodd *et al*., 2005). Unlike animals, plants are unable to move on the ground. However, their leaves can respond to environmental stimulations such as light, temperature, touching and chemical substances by visible movement (McClung, 2006; Ueda & Nakamura, 2007).

Many plants fold their leaves at night. Such nyctinastic movements are common in the legume family (Leguminoseae) and the wood sorrel family (Oxalidaceae). Nyctinastic leaf movements are regulated by a circadian clock with a cycle of *c*. 24 h (Ueda & Nakamura, 2007). Although the phenomenon was observed in ancient times, it was Darwin who studied and illustrated leaf movements in various plants including *Medicago* (Darwin, 1880). Videos of such movement can now be easily found on the internet, and people are still fascinated with it. Despite the long history of observing and studying this interesting phenomenon, the molecular basis and functional reason behind nyctinasty are still unknown.

Structurally, nyctinastic movement is mediated by the pulvinus, which is a specialized motor organ located at the base of leaves and leaflets. Two functionally different tissues, the adaxial flexor and the abaxial extensor, constitute the pulvinus (Uehlein & Kaldenhoff, 2008). The oscillations in leaf movement are generated by rhythmic swelling and shrinking of the motor cells of pulvini. Therefore, the pulvinus plays a key role in leaf movement.

Several mutants of legume species with defects in pulvini have been reported, such as the *apulvinic* mutant in *Pisum sativum* (Marx, 1987) and the *sleepless* mutant in *Lotus japonicus* (Kawaguchi, 2003). These mutants lacked pulvini and formed petiole-like structures at the base of leaflets. However, the gene responsible for pulvinus development is unknown. In addition, it is unclear whether such leaf movement may confer any advantage for plant growth.

*Medicago truncatula* is a model legume species that undergoes nyctinastic movement by folding its leaflets from horizontal to vertical. In recent years, a *Tnt1* retrotransposon-tagged *M. truncatula* mutant population was generated (Tadege *et al*., 2008) and a number of mutants affecting leaf patterning and development were identified and characterized. It has been documented that multiple genes are involved in leaf pattern identification in *M. truncatula*, including *PALMATE-LIKE PENTAFOLIATA1* (*PALM1*), *SINGLE LEAFLET1* (*SGL1*) and *Mt NO APICAL MERISTEM* (*MtNAM*) (Wang *et al*., 2008; Chen *et al*., 2010; Cheng *et al*., 2012). The *palm1*, *sgl1* and *mtnam* mutants exhibit pentafoliate leaves, simple leaves and fused leaves, respectively. In addition, compound leaf formation requires the definition of leaflet boundaries. *LATERALORGAN BOUNDARIES* (*LOB*) is a gene that is expressed specifically in organ boundaries. In *Arabidopsis*, however, the *lob* mutant does not display obvious phenotype as a result of functional redundancy of other gene members in this family (Shuai *et al*., 2002). The role of *LOB* in compound-leafed species remains undetermined.

In this study, mutants with defects in leaf movement were isolated from the *Tnt1* retrotransposon-tagged mutant population of *M. truncatula*. Loss of function in *PETIOLULE-LIKE PULVINUS (PLP)* leads to the change of pulvini to petiolules. The gene was cloned and molecular analysis showed that PLP is a unique *LOB* domain protein. The specific spatial and temporal expression pattern of *PLP* suggests that PLP plays a key role in the development of pulvini. Global transcript profiling analysis revealed that loss of function of *PLP* affects the expression of a large number of genes during both day and night. Comparison of biomass yield between wild-type and *plp* mutants indicates that leaf movement may have an impact on plant vegetative growth.

## Materials and Methods

### Plant material and growth conditions

*Medicago truncatula* Gaertn. Ecotype R108 was used as the wild type for all the experiments described in this study. Plants were grown in Metro-Mix 830 soil mix at 22°C day/20°C night temperature, 16-h day/8-h night photoperiod and 70–80% relative humidity. The four *plp* alleles identified were: NF2623 (*plp-1*, ecotype R108; a *Tnt1* inserted between 178 bp and 179 bp), NF0571 (*plp-2*, ecotype R108; a *Tnt1* inserted between 130 bp and 131 bp), F9359-LTR4 (*plp-3*, ecotype A17; a *Tnt1* inserted between 36 bp and 37 bp of *PLP*) and NF5514 (*plp-4*, ecotype R108; a *Mere1* inserted between 246 bp and 247 bp).

### Molecular cloning of *PLP* and vector construction

The coding sequence of *PLP* was amplified by polymerase chain reaction (PCR) from cDNA of wild-type *M. truncatula* and deposited in GenBank (JN412594). The primers used for cloning of *PLP* were: PLP-CDS-F, CACCATGGCATCATCAAGCTCATAC; and PLP-CDS-R, TCACAAATTACCTCCTCCTACA. To functionally complement the *plp* mutant, a 2414 bp *PLP* promoter sequence plus a 1611 bp *PLP* genomic DNA sequence were PCR-amplified and cloned into the pENTR™/D-TOPO cloning vector (Invitrogen, Chicago, IL, USA). Then the fragment was transferred into the pEarleyGate 301 vector (Earley *et al*., 2006) by attL × attR recombination reactions (Invitrogen). The following primers were used to amplify the *PLP* promoter and the genomic DNA sequences: PLP-Com-F: CACCGTTAAAGTGTATCATAAGGGAG and PLP-Com-R:TCACTGCTCGTTTTCCTGTTAG.

### β-Glucuronidase (GUS) staining and microscopy

Fully expanded leaves were collected for GUS staining. The GUS activity was histochemically detected as previously described (Jefferson *et al*., 1987). For scanning electron microscopy, tissue samples were fixed in 3% (v/v) glutaraldehyde and then dehydrated in a series of ethanol. The samples were observed using Hitachi TM-3000 SEM at an accelerating voltage of 15 kV. For fluorescent imaging, a Leica TCS SP2 AOBS confocal laser scanning microscope was used. The 488-nm line of an argon laser was chosen for the green fluorescent protein (GFP) signal, and emission was detected at 150 nm.

### Plant transformation

The final binary vectors were transferred into the disarmed *Agrobacterium tumefaciens* strain EHA105 using the freezing/heat shock method. Leaves of wild-type and *plp-1* were transformed with EHA105 harboring various vectors (Cosson *et al*., 2006).

### RNA extraction, RT-PCR, qRT-PCR and microarray analysis

Leaves of 4-wk-old wild-type and *plp* mutant plants were collected for RNA isolation. Total RNA was isolated using Trizol Reagent (Invitrogen). One microgram of RNA was reverse transcribed with SuperscriptIII (Invitrogen) following the manufacturer's instructions. The cDNA was used as a template for reverse transcription PCR (RT-PCR) and quantitative RT-PCR (qRT-PCR). Primers used for quantifying the expression level of *PLP* were PLP-qPCR-F (ATCACAAACAGCGCAGGAGAA) and PLP-qPCR-R(GGCTGCACATGGTGAATTGTAT). For microarray analysis, total RNA was extracted from three biological replicates of fully expanded leaves of 4-wk-old *plp-1* mutant and wild-type-like plants in a segregating F_2_ population. RNA purification, probe labeling, hybridization, and scanning for microarray analysis were conducted as previously described (Zhou *et al*., 2011a). Functional enrichments were calculated with PageMan (Usadel *et al*., 2006) and visualized with mapman (Thimm *et al*., 2004).

### *In situ* hybridization analysis

A fragment of 510 bp *PLP* coding sequence (CDS) was amplified by PCR using primers PLP-is-F: TTGCACCATACTTTCCACCGGA and PLP-is-R: ACTTCTTCTGTCACCAGTGCCT. The PCR product was labeled with Digoxigenin (Digoxigenin-11-UTP, Roche Diagnostics). RNA *in situ* hybridization was performed on shoot apices or inflorescence apices of 4-wk-old wild-type plants as previously described (Zhou *et al*., 2011b).

### Phylogenetic tree

Full-length protein sequences of PLP and other lateral organ boundary (LOB) members were used for phylogenetic analysis. Alignments were performed using jalview with default parameters (Waterhouse *et al*., 2009). Phylogenetic tree was constructed by the mega4 program (Tamura *et al*., #b^300^) with 1000 bootstrap replications.

## Results

### *PLP* is required for pulvinus development

**Fig. 1:**
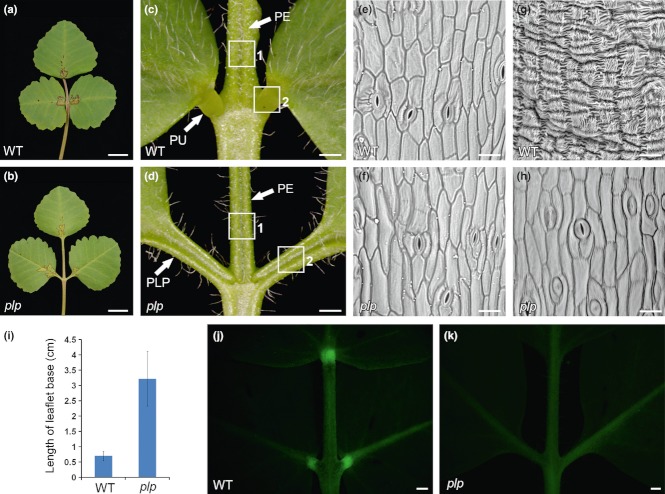
The *petiolule-like pulvinus* (*plp*) mutant of *Medicago truncatula* shows developmental defects in pulvini. (a,b) Adult leaf of wild type and *plp* mutant. (c,d) Close-up view of leaflet base in wild-type and *plp* mutant. (e,f) Scanning electron micrograph of epidermal cells in petiolule of wild type (box 1 in (c)) and *plp* mutant (box 1 in (d)). (g,h) Scanning electron micrograph of epidermal cells in pulvinus of wild type (box 2 in (c)) and petiolule-like pulvinus of *plp* mutant (box 2 in (d)). (i) Length of lateral leaflet base of wild type and *plp* mutant. Values are means ± SD (*n* = 20). (j,k) *DR5rev*:*green fluorescent protein (GFP)* expression in the leaf (abaxial side) of wild type and *plp* mutant. PE, petiolule; PU, pulvinus; PLP, petiolule-like pulvinus; Bars, (a,b) 5 mm; (c,d,j,k) 500 μm; (e–h) 20 μm.

The adult leaves of *M. truncatula* are in trifoliate form consisting of one terminal leaflet and two lateral leaflets. Each leaflet has a pulvinus that is responsible for leaflet movement. To identify mutants with defects in leaf movement, a large number (*c*. 10 000) of *Tnt1* retrotransposon-tagged *M. truncatula* lines were screened. Four independent mutant lines with obvious defects in leaf movement were identified. In contrast to the wild-type, the pulvini developed in the mutants were changed to petiolule-like structures (Fig. 1a–d). Therefore, the mutation was named *petiolule-like pulvinus* (*plp*) and the allelic lines were designated *plp-1*, *plp-2*, *plp-3*, and *plp-4*.

Scanning electron microscopy (SEM) analysis was performed to characterize the defects of the *plp* mutants. While the epidermal cells of petiolules in wild-type and mutants showed similar shape (Fig. 1e,f), the shape of the epidermal cells of pulvini in wild-type and mutants were different (Fig. 1g,h). The pulvini of the wild-type consisted of specific motor cells (Fig. 1g). However, cells of petiolule-like pulvini in *plp* mutants were elongated and the stomata were observable, similar to the epidermal cells of petiolule (Fig. 1h). Accompanying the changes in pulvini was an increase in the leaflet base length in the *plp* mutants (Fig. 1i). These observations indicate that loss of function in *PLP* leads to defects in the determination of pulvinus cell fate, resulting in the development of petiolule-like pulvinus in the *plp* mutants. A previous study showed that auxin plays important roles in various organ development in *M. truncatula* (Zhou *et al*., 2011b). To test if auxin accumulation is associated with pulvinus development, the *DR5rev:GFP* auxin response reporter was introduced into the wild-type and *plp-1* mutant, respectively. A GFP signal was detected specifically in the pulvini, but not in petiolules of wild type (Fig. 1j). In contrast, no obvious auxin accumulation was observed at the same location in the *plp* mutants (Fig. 1k). This observation further suggests that the pulvini lost their developmental characteristics and were changed to petiolules in the *plp* mutants.

### *PLP* encodes a LOB domain protein

**Fig. 2:**
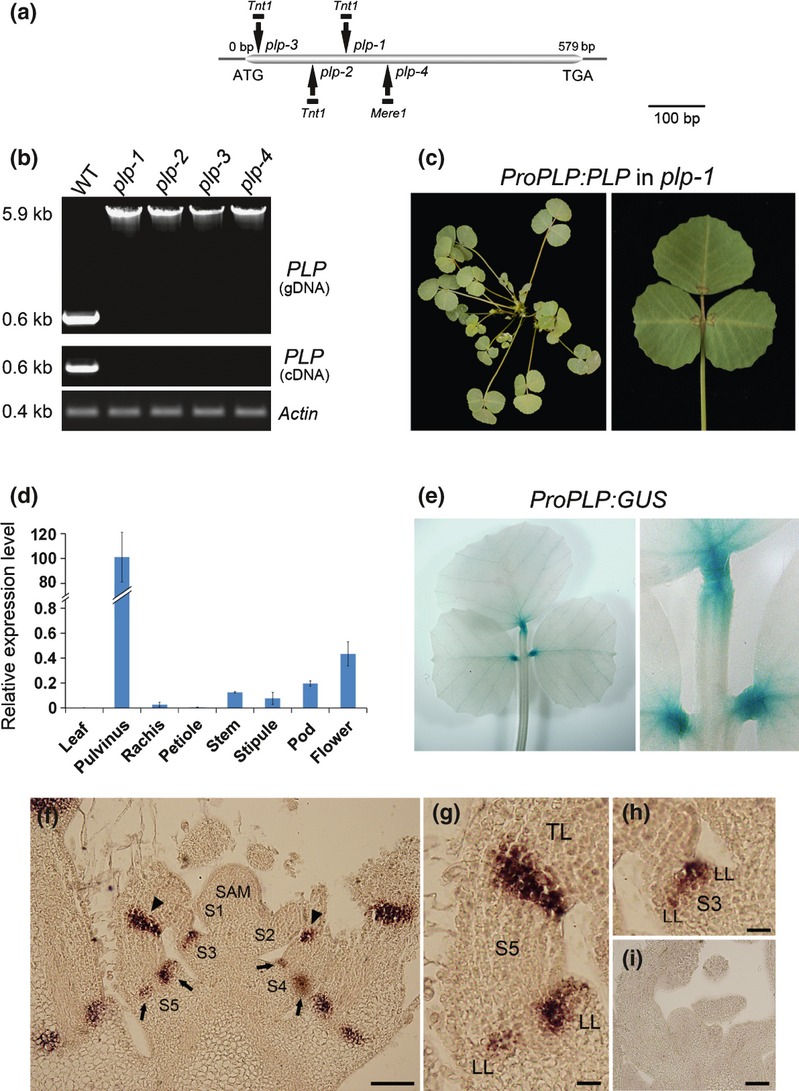
Molecular cloning and expression pattern of *PLP* in *Medicago truncatula*. (a) Schematic representation of the gene structure of *PLP*. The positions of ATG start and TGA stop codons are shown. Vertical arrows mark the location of *Tnt1* or *Mere1* retrotransposons in *plp* alleles. (b) PCR and RT-PCR amplification of *PLP* from the wild-type (WT) and *plp* mutants. A single insertion (*c*. 5.3 kb) was detected in each mutant line. *PLP* expression was not detected in the mutants. *Actin* was used as a loading control. Three technical replicates were performed. (c) Genetic complementation of the *plp* mutant. A representative *plp-1* line transformed with the *PLP* genomic sequence (*ProPLP : PLP*) showed normal wild-type-like leaves and pulvini. (d) Quantitative reverse transcription-polymerase chain reaction (qRT-PCR) analysis of the expression pattern of *PLP*. Values are means ± SD (*n* = 3). (e) β-Glucuronidase (GUS) staining of trifoliates of transgenic *M. truncatula* plants carrying the *PLP* Promoter-GUS construct. (f–i) *In situ* hybridization analysis of *PLP* mRNA in vegetative apices of the wild type. Longitudinal section of the shoot apical meristem (SAM) is shown in (f). Arrows point to lateral leaflet base. Arrowheads point to terminal leaflet base. The close-up views of S5 and S3 are shown in (g) and (h), respectively. (i) *In situ* hybridization using the sense probe as control. S1–S5, stage1 to stage5; TL, terminal leaflet primordia; LL, lateral leaflet primordia. Bars, (f,i) 50 μm; (g,h) 10 μm.

To identify the gene responsible for the mutant phenotype, thermal asymmetric interlaced-PCR (TAIL-PCR) was performed to recover the flanking sequences from *plp-1*. In total, 16 flanking sequences were identified in *plp-1*, and one of the sequences was associated with the mutant phenotype. A full-length genomic sequence was obtained after using the flanking sequence to search against the *M. truncatula* genome from the National Center for Biotechnology Information. The 579-nucleotide full-length CDS of this gene was cloned by RT-PCR. Alignment between the cDNA and genomic sequences revealed that *PLP* contains one exon (Fig. 2a). The *Tnt1* insertion in *plp-1* was located in the exon. Further sequence analysis revealed that the other three mutants carried either a tobacco *Tnt1* retrotransposon or a *M. truncatula Mere1* retrotransposon (Rakocevic *et al*., 2009) in the exon of *PLP*. The results were confirmed by genomic PCR analysis (Fig. 2b). Reverse transcription-PCR was performed and no transcripts of *PLP* were detected in the four alleles (Fig. 2b). To further confirm that the mutant phenotype was caused by disruption of this gene, a construct carrying a 4.2-kb genomic sequence, including a 2.5-kb promoter region and the *PLP* open reading frame, was introduced into homozygous *plp-1* mutant plants. Phenotypic observation showed that the defects in pulvini were fully rescued (Fig. 2c).

Sequence alignment was performed between PLP and its putative orthologs from *Glycine max*, *Vitis vinifera*, *Lotus japonicus*, *Zea mays*, *Triticum aestivum* and *Arabidopsis thaliana*. The results showed that PLP contains a highly conserved LOB domain (see the Supporting Information, Fig. S1). Phylogenetic analysis with 42 members of the LOB domain gene family from *Arabidopsis* revealed that PLP was evolutionarily closer to ASL4/LOB (At5g63090) and showed 75% identity with ASL4/LOB (Fig. S2).

### *PLP* specifically expresses in pulvini

The expression pattern of *PLP* from the *M. truncatula* Gene Expression Atlas showed a relatively high expression level of *PLP* in vegetative buds (Fig. S3). To compare the expression level of *PLP* in pulvini and other tissues, qRT-PCR was performed. The data revealed that *PLP* was highly expressed in the pulvinus compared with other plant organs (Fig. 2d). To confirm this specific expression pattern, a *PLP* promoter driven β-glucuronidase (GUS) reporter gene was introduced into wild-type plants. The expression of GUS was mainly detected in the pulvini of transgenic plants (Fig. 2e). These results suggest that PLP plays a highly specific role in the development of pulvinus.

In *M. truncatula*, it has been shown that lateral leaflet primordia and the terminal leaflet primordium develop at different stages (Wang *et al*., 2008). A pair of lateral leaflet primordia (LL) is developed first at the proximal end of the common leaf primordium, followed by the development of the terminal leaflet primordium (Wang *et al*., 2008). The development of both terminal and lateral leaflets is associated with the formation of pulvini. To explore the spatial and temporal localizations of *PLP* during the development of pulvini, RNA *in situ* hybridization was performed in the wild type (Fig. 2f–i). *PLP* mRNA was first detected in the leaf primordium at stage 3 (S3) in which lateral leaflet primordia were developing (Fig. 2h). At stage 4 (S4), three strong hybridization signals were detected in leaf primordium where the pulvini were formed. Two of them were associated with the lateral leaflets (Fig. 2f, arrows) and one was associated with the terminal leaflet (Fig. 2f, arrowheads). This elaborate expression pattern was continually exhibited in the older leaf primordia (Fig. 2g). The specific spatial and temporal expression pattern of *PLP* further demonstrates that PLP is tightly associated with the development of pulvini.

### Genetic interactions between *plp* and leaf pattern mutants

**Fig. 3:**
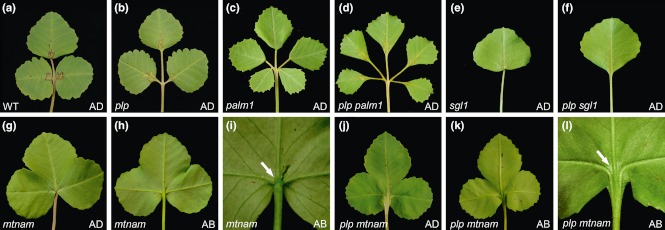
Genetic interactions between *plp* and different leaf pattern mutants. Adult leaves of wild-type *Medicago truncatula* and the mutants are shown. (a) Wild type; (b) *plp* mutant; (c) *palm1* mutant; (d) *plp palm1* double mutant; (e) *sgl1* mutant; (f) *plp sgl1* double mutant; (g,h) *mtnam* mutant; (i) close-up view of leaf base of the *mtnam* mutant, the arrow points to the clustered pulvini; (j,k) *plp mtnam* double mutant; (l) close-up view of leaf base in the *plp mtnam* double mutant, the arrow points to the separated petiolule-like pulvini. AD, adaxial side; AB, abaxial side.

Defects in the pulvini of *plp* mutants suggest that *PLP* is required for the proper formation of leaves. To investigate the possible role of *PLP* in leaf patterning, the *plp* mutant was crossed with the leaf pattern mutants *palm1*, *sgl1* and *mtnam*. Compared with the phenotype of wild type and single mutants, the *plp palm1* double mutant exhibited pentafoliolate leaves with petiolule-like pulvini (Fig. 3a–d). The *plp sgl1* double mutant had simple leaves, resembling those of *sgl1* single mutant. The pulvini of *plp sgl1* were similar to those in the *plp* single mutant (Fig. 3e–f). The weak allele of *mtnam* was used for generating a double mutant because the strong *mtnam* allele was seedling-lethal. In *mtnam*, the midveins of lateral leaflets were fused with the midvein of the terminal leaflet (Fig. 3g–i). The *plp mtnam* double mutant also showed fused leaflets. However, elongation of the proximal end of midveins was observed, indicating that the pulvini were also changed to petiolules in the double mutant. In addition, clustered pulvini were observed in *mtnam* (Fig. 3i, arrow) while separated petiolule-like pulvini were found in *plp mtnam* (Fig. 3l, arrow), indicating that *MtNAM* and *PLP* may play different roles in organ separation and boundary identification. These observations suggest that the developmental mechanism of pulvini determination and leaf pattern are probably independent. The findings further imply that the pulvini are tightly associated with leaflet formation irrespective of changes in leaflet number and pattern.

### Global changes in gene expression in *plp* mutant

**Fig. 4:**
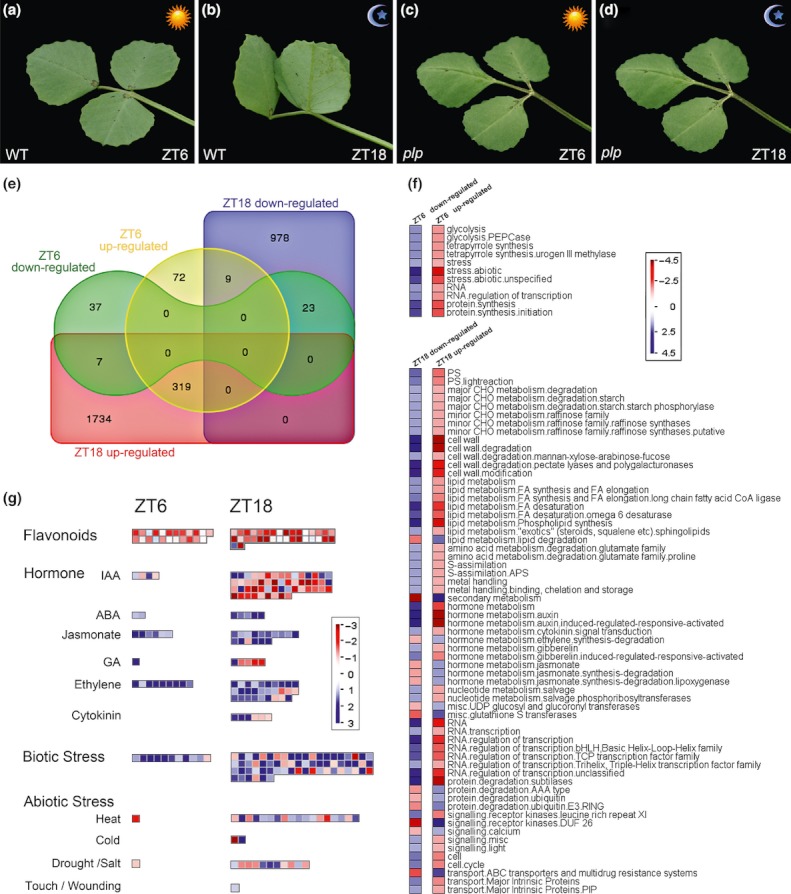
Leaf movement and differential expression of metabolic genes in *Medicago truncatula* wild-type and *plp* mutant leaves at ZT6 (noon) and ZT18 (midnight). (a,b) Leaf movement of wild type (WT) at ZT6 and ZT18. (c,d) The *plp* mutant lost its usual dynamic movement of leaflets because of the altered structure of pulvinus. (e) Venn diagram (http://bioinformatics.psb.ugent.be/webtools/Venn/) of total numbers of upregulated and downregulated genes in the *plp* mutant at ZT6 and ZT18. (f) PageMan functional enrichments of differentially expressed genes. Red, underrepresented functional categories; blue, overrepresented functional categories; (g) Selected significantly differentially expressed genes in flavonoids and hormone metabolism, as well as in biotic and abiotic stress. Visualization of these genes with MapMan 3.5.1R2 using log2-transformed ratios of the mutant divided by wild-type. Red, up-regulated genes; blue, down-regulated genes.

The leaflets of *M. truncatula* exhibit nyctinastic movement with circadian rhythms. They are usually horizontal (open) during the day and vertical (folded or closed) at night (Fig. 4a,b). The closed leaflets start to move becoming horizontal at dawn (06:00 h, Zeitgeber time 0, ZT0). The opened leaflets start to move vertically at 19:00 h (ZT13) and continue the movement until *c*. 21:00 h (ZT15) to close completely. In *plp* mutants, the leaflets lost their usual dynamic movement because of the altered structure of pulvini (Fig. 4c,d). To investigate molecular response to *PLP* associated leaf movement, gene transcript levels in wild type and *plp-1* mutant were measured using AffymetrixMedicagoGenechips (Affymetrix, CA, USA). Fully expanded leaves were collected from plants at noon (ZT6) and at midnight (ZT18) and used for RNA extraction and microarray analysis.

Compared with the wild type, the expression levels of 467 genes at ZT6 and of 3070 genes at ZT18 were altered by at least twofold in the *plp* mutant (see the Supporting Information, Tables S1, S2). Among 400 upregulated and 67 downregulated genes in *plp* mutants at ZT6, only nine and seven genes were oppositely regulated at ZT18, respectively, suggesting that abolished leaf movement associated with *PLP* affects gene expression in a consistent manner during both day and night (Fig. 4e). To characterize the possible functions of differentially expressed genes, functional enrichment was analysed by PageMan (Usadel *et al*., 2006). The results revealed that upregulated genes at ZT18 were enriched in lipid metabolism, secondary metabolism, hormone metabolism, protein degradation and transport. The upregulated genes at ZT6 were under-represented in all functional categories. However, most of the downregulated genes at both ZT6 and ZT18 were over-represented. They were enriched for genes involved in photosynthesis, stress, protein synthesis, cell wall degradation, lipid metabolism, hormone metabolism and RNA transcription. The data suggests that broad effects in multiple biological processes were induced by *PLP* associated leaf movement.

To further illustrate the effects of *PLP*-associated leaf movement on plant growth and development, differentially expressed genes at ZT6 and ZT18 were mapped onto MapMan (Fig. S4, metabolism overview; Tables S3, S4) (Thimm *et al*., 2004). The data showed that changes in the amount of gene transcript were observed in most metabolic processes. Among these changes, genes involved in secondary metabolism of terpenes, flavonoids and phenylpropanoids/phenolics were strongly upregulated at ZT18. It is noted that the flavonoid-related genes are induced at both ZT6 and ZT18, suggesting that flavonoid metabolism was affected in *plp* mutants both during the day and at night (Fig. 4g). Further analysis showed that genes involved in hormone metabolism were changed dramatically at ZT18 compared with those at ZT6 (Fig. 4g). In addition, the expression levels of both biotic and abiotic stress-related genes were changed at both time-points, especially at ZT18. These observations imply that loss of function of *PLP* probably has negative effects on their growth. To verify this hypothesis, the vegetative growth of 3-wk-old plants of wild type and *plp* mutants was analysed. The projected leaf area of 48 plants from wild-type and *plp* mutant was calculated (Fig. S5a,b). The data showed that wild-type plants had 15% more projected leaf area than that of the mutants (Fig. S5c). Furthermore, biomass yield of the aboveground parts of wild-type plants was significantly higher than the yield of mutant plants (Fig. S5d).

## Discussion

In this study, four independent *PLP* alleles were found. These alleles showed the same defects in the determination of developmental identity of pulvini. *PLP* is identified as a member of the plant-specific LOB domain gene family in *M. truncatula*. In *Arabidopsis*, the *LOB* gene is expressed at the base of all lateral organs. The expression pattern indicates that it may play a crucial role in defining organ boundaries (Shuai *et al*., 2002; Majer & Hochholdinger, 2011). In *M. truncatula*, pulvini are formed when the leaflet primordia emerge and localize at the joint between leaflet and petiole. Therefore, the formation of pulvini defines of boundaries between leaflet and petiole. In compound-leafed species, loss of function in boundary formation-related genes resulted in fused leaves (Berger *et al*., 2009; Cheng *et al*., 2012). Unlike those genes, *PLP* defines the specific boundaries between leaflet and petiole by playing a unique role in the development of pulvini, as revealed by the following lines of evidence. First, the pulvinous differentiation program did not proceed because of the absence of *PLP*. The pulvini in the *plp* mutants were changed to petiolules with elongated epidermal cells and increased organ length. In addition, auxin accumulation was specifically observed in the pulvini of adult leaves in wild type, while no such auxin accumulation was detected in the *plp* mutants, further confirming that the unique structure of pulvini was altered in the mutant. Second, *PLP* was highly expressed in the pulvini, supported by the qRT-PCR data and the analysis of transgenics carrying the *PLP* promoter–GUS reporter construct. Furthermore, the developmental link between *PLP* expression and pulvini emergence was revealed by *in situ* hybridization. Third, genetic interactions between *plp* and leaf pattern mutants *sgl1* and *palm1* indicate that PLP is not involved in the determination of leaf pattern but is associated with leaflet formation. The weak allele of *mtnam* was used to produce the double mutant with *plp*. Fused leaflets but separated petiolule-like pulvini in the double mutant suggest that *MtNAM* and *PLP* may regulate the formation of different boundaries. In this proposed scenario, *MtNAM* plays a conserved role in boundary identification between leaflets (Blein *et al*., 2008; Cheng *et al*., 2012) while *PLP* specifically elaborates boundary formation between leaflet and petiole through activation of the pulvinous differentiation program. Such a program is required for the identification of pulvini where compact and convoluted motor cells are produced. The loss of cell fate identity in the petiolule-like pulvinus of *plp* led to the development of elongated cells similar to those in rachis. Therefore, the formation of elongated petiolules in the *plp* mutant results from the conversion of convoluted motor cells to regular rachis cells. It has been reported that conserved gene modules function in a context-dependent manner (Efroni *et al*., 2010; Hay & Tsiantis, 2010). Petiolules are present in most compound leafed species, but pulvini only exist in some species with nyctinastic movement. This phenomenon suggests that pulvini development is the result of context-specific effects and that the conserved *LOB* genes are differentially deployed among species, resulting in morphological diversity. We propose that the formation of boundaries regulated by PLP is required for plant species that are capable of displaying nyctinastic leaf movement.

Leaf movement may have particular functional significance for plant growth by optimizing light intensity on leaf surface during the day. It has been suggested that nyctinastic leaf movement could be a type of self-adjusting mechanism in response to environmental changes. In order to better understand the biological effects of leaf movement, we analysed global changes of gene expression in *plp-1* mutant during the day and at night. The microarray experiments revealed that transcriptional changes occur at both ZT6 and ZT18. Surprisingly, the number of upregulated or downregulated genes at ZT18 was about seven times more than that at ZT6, suggesting that loss of function of *PLP* has a large impact on gene expression at night. It seems that a dynamic gene regulatory mechanism exists even after the closing of leaflets and without light stimulation. Further research is needed to explore this area.

Functional enrichment analysis revealed overrepresentation of genes at ZT18 involved in multiple biological processes, suggesting that loss of function of *PLP* has broad effects on plants. Previous reports showed that flavonoid accumulation is associated with the biotic or abiotic stress conditions (Christie *et al*., 1994; Dixon & Paiva, 1995; Vanderauwera *et al*., 2005; Hernandez & Van Breusegem, 2010; Kang *et al*., 2011). In addition, flavonoids also protect leaves from light stress and UV-B radiation (Gould *et al*., 2000; Jordan, 2002; Deavours & Dixon, 2005). The upregulation of genes involved in flavonoid metabolism in the *plp* mutant indicate that the mutant plants may undergo certain stresses. This hypothesis is supported by the expression changes of genes involved in biotic stress and abiotic stress. The expression level of hormone metabolism-related genes are also altered in mutants. The comprehensive changes in hormone metabolism are probably the result of crosstalk between plant hormones in response to biotic and abiotic stresses (Spoel & Dong, 2008; Wolters & Jurgens, 2009; Robert-Seilaniantz *et al*., 2011). Consistent with these findings, wild-type plants showed a larger leaf area and higher biomass yield than those of the mutants, suggesting that nyctinastic leaf movement associated with *PLP* may have an important impact on plant vegetative growth.

In summary, we identified *plp* mutants with defects in leaf movement. The specific function of *PLP* was characterized in detail. *PLP* encodes a unique LOB domain protein and plays a crucial role in the determination of pulvinus development. Nyctinastic leaf movement associated with *PLP* greatly impacts on gene expression and may benefit plant growth and development.

## Supporting Information

**Fig. S1** Alignment of amino acid sequences of PLP and its putative orthologs.

**Fig. S2** Phylogenetic tree of PETIOLULE-LIKE PULVINUS (PLP) and members of the lateral organ boundary (LOB) domain gene family from *Arabidopsis*.

**Fig. S3** Expression profiling of *PLP* transcripts.

**Fig. S4** Differential expression of metabolic genes in the *plp* mutant.

**Fig. S5** Vegetative growth of wild-type and *plp* mutant plants.

**Table S1** List of downregulated and upregulated genes/probe sets in the *plp* mutant at ZT6

**Table S2** List of downregulated and upregulated genes/probe sets in the *plp* mutant at ZT18

**Table S3** List of genes involved in flavonoid metabolism, hormone metabolism and stress response at ZT6

**Table S4** List of genes involved in flavonoid metabolism, hormone metabolism and stress response at ZT18
