# Supplementary material for: Identification and characterization of petiolule-like pulvinus mutants with abolished nyctinastic leaf movement in the model legume Medicago truncatula
Source: New Phytol. 2012 Aug 14;196(1):92–100. doi: 10.1111/j.1469-8137.2012.04268.x (PMC3504090; doi:10.1111/j.1469-8137.2012.04268.x)
Supplement: Supplementary file 1 [file nph0196-0092-SD1.pdf]

Supporting Information Figs S1-S5

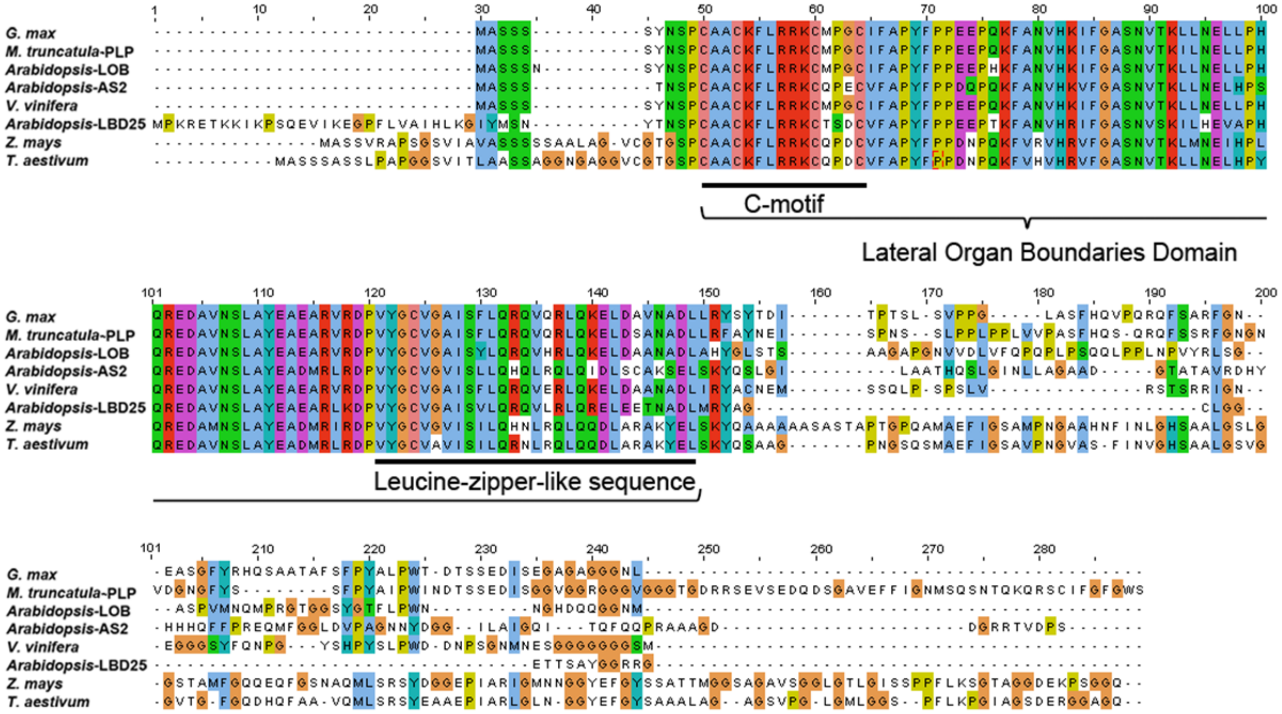

Fig. S1 Alignment of amino acid sequences of PLP and other LOB proteins from *Glycine max*, *Vitis vinifera*, *Zea mays*, *Triticum aestivum* and *Arabidopsis*. The frame shows the conserved lateral organ boundary domain.

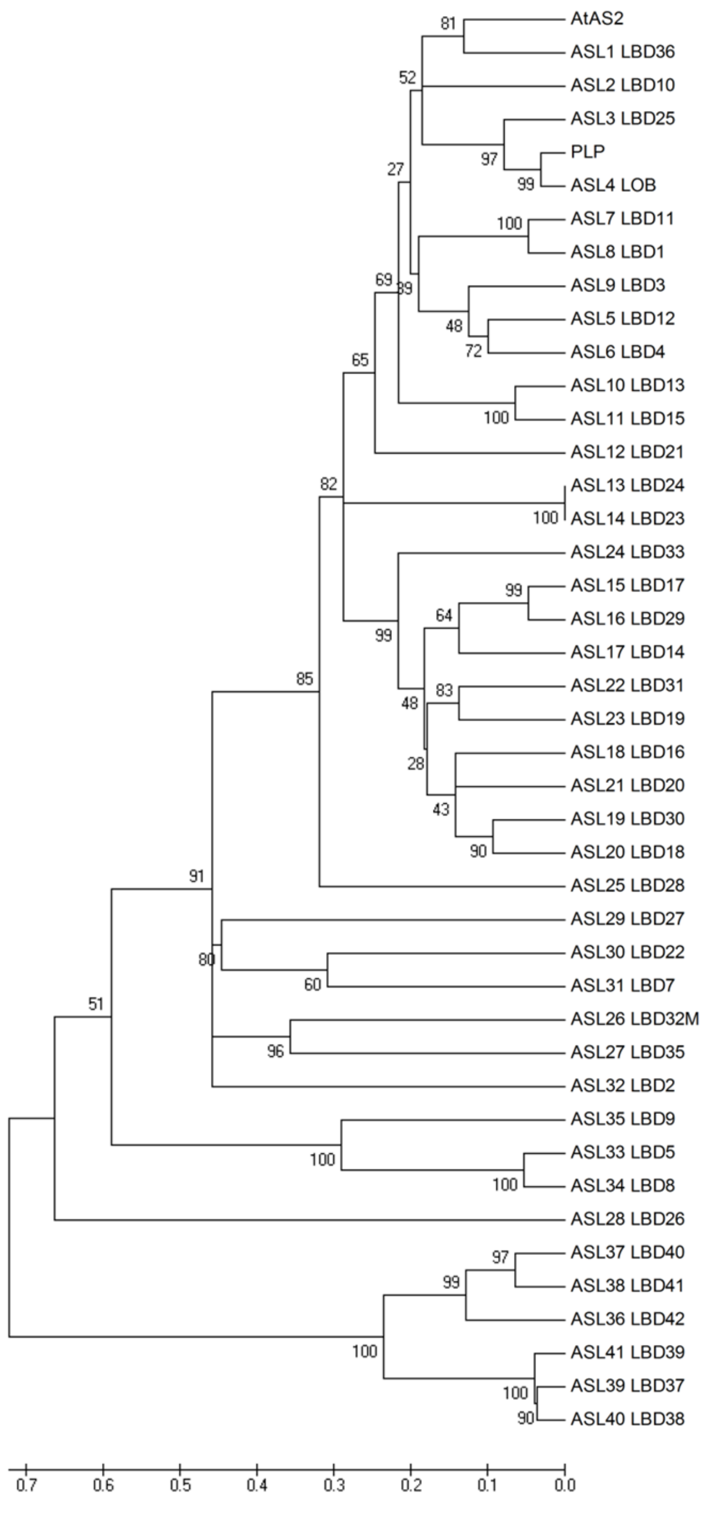

Fig. S2 Phylogenetic tree of PLP and other members of the LOB domain gene family from *Arabidopsis*. PLP is evolutionarily closer to the ASL4/LOB (At5g63090).

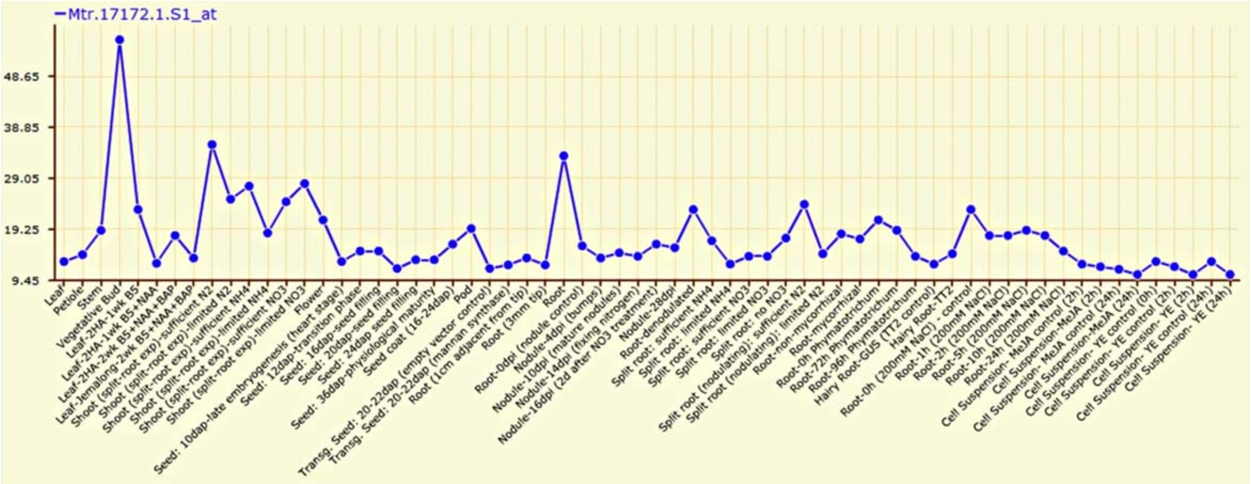

Fig. S3 Expression profiling of the *PLP* transcripts.

The data for *PLP* (probe set *Mtr.17172.1.S1_at*) is accessible at http://bioinfo.noble.org/gene-atlas/.

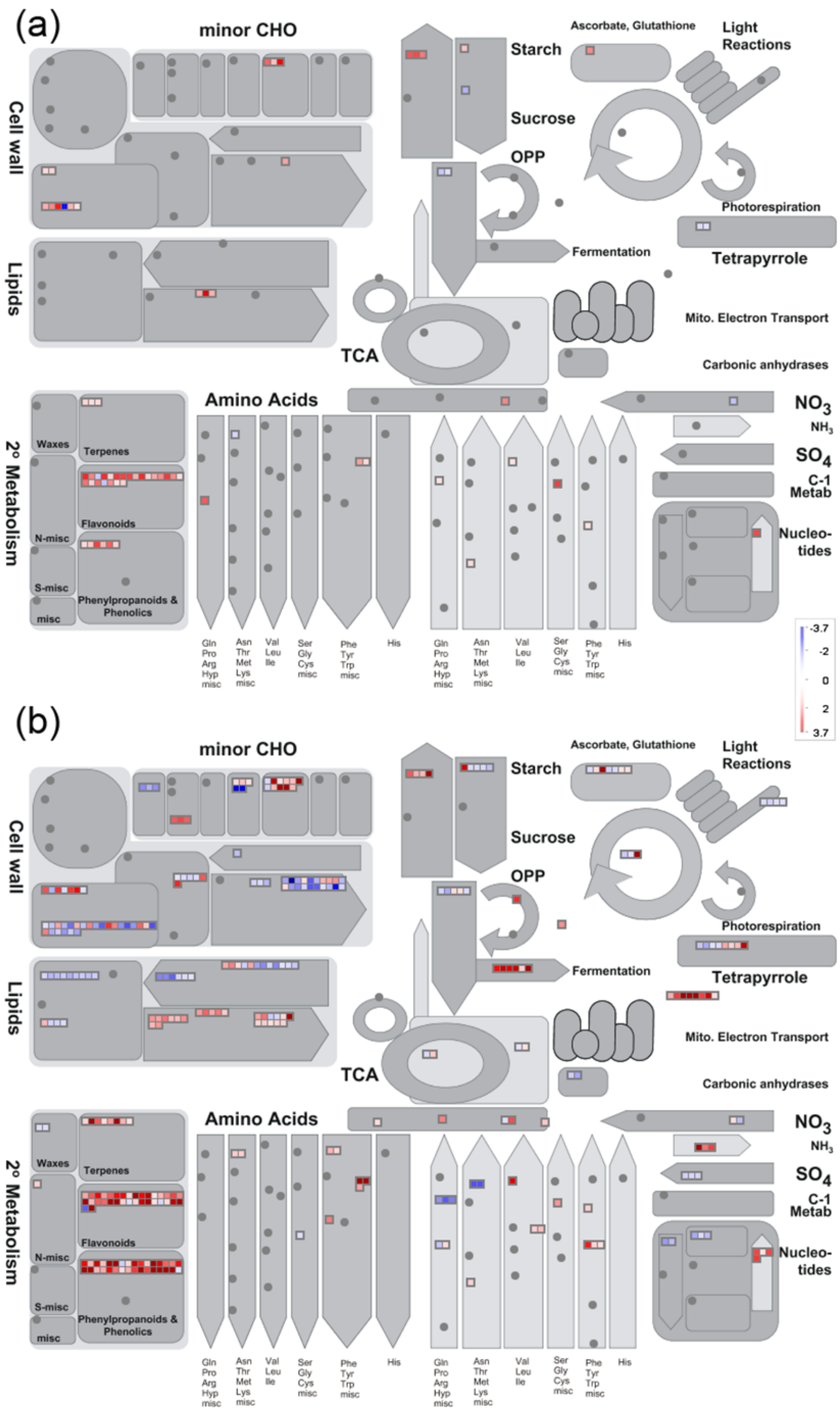

Fig. S4 Differential expression of metabolic genes in the *plp* mutant.

Visualization of the genes at ZT6 (a) and ZT18 (b) with MapMan 3.5.1R2 using log2-transformed ratios of mutant divided by wild-type. Red, up-regulated genes; Blue, down-regulated genes.

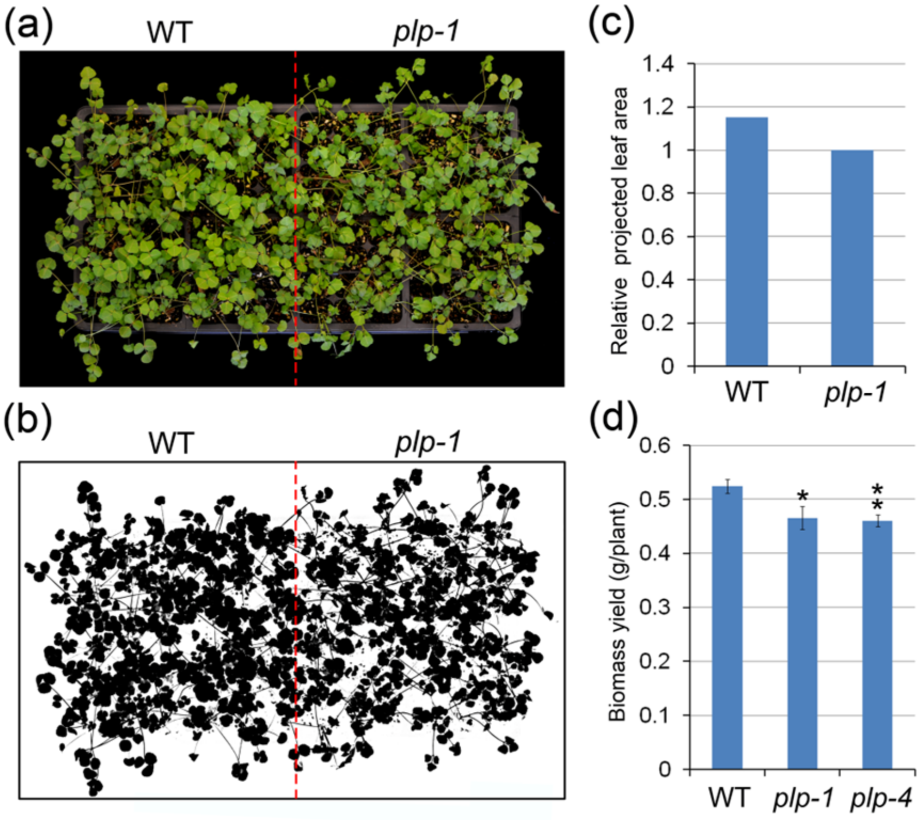

Fig. S5 Vegetative growth of wild-type and *plp* mutant plants.

(a) 48 three-week-old plants of wild-type (left) and *plp-1* mutant (right) are growing in a single tray. (b) Image of projected leaf area of wild-type and *plp-1* mutant plants in the same tray. (c) Relative projected leaf area of wild-type and *plp-1* mutant. (d) Average of fresh biomass yield of above ground part of wild-type, *plp-1* and *plp-4* mutant plants. Values are means ± SE (n = 80). *P*-values: *, < 0.05; **, < 0.005.
