# Supplementary figures and images for: Identification and characterization of petiolule-like pulvinus mutants with abolished nyctinastic leaf movement in the model legume Medicago truncatula
Source: New Phytol. 2012 Aug 14;196(1):92–100. doi: 10.1111/j.1469-8137.2012.04268.x (PMC3504090; doi:10.1111/j.1469-8137.2012.04268.x)

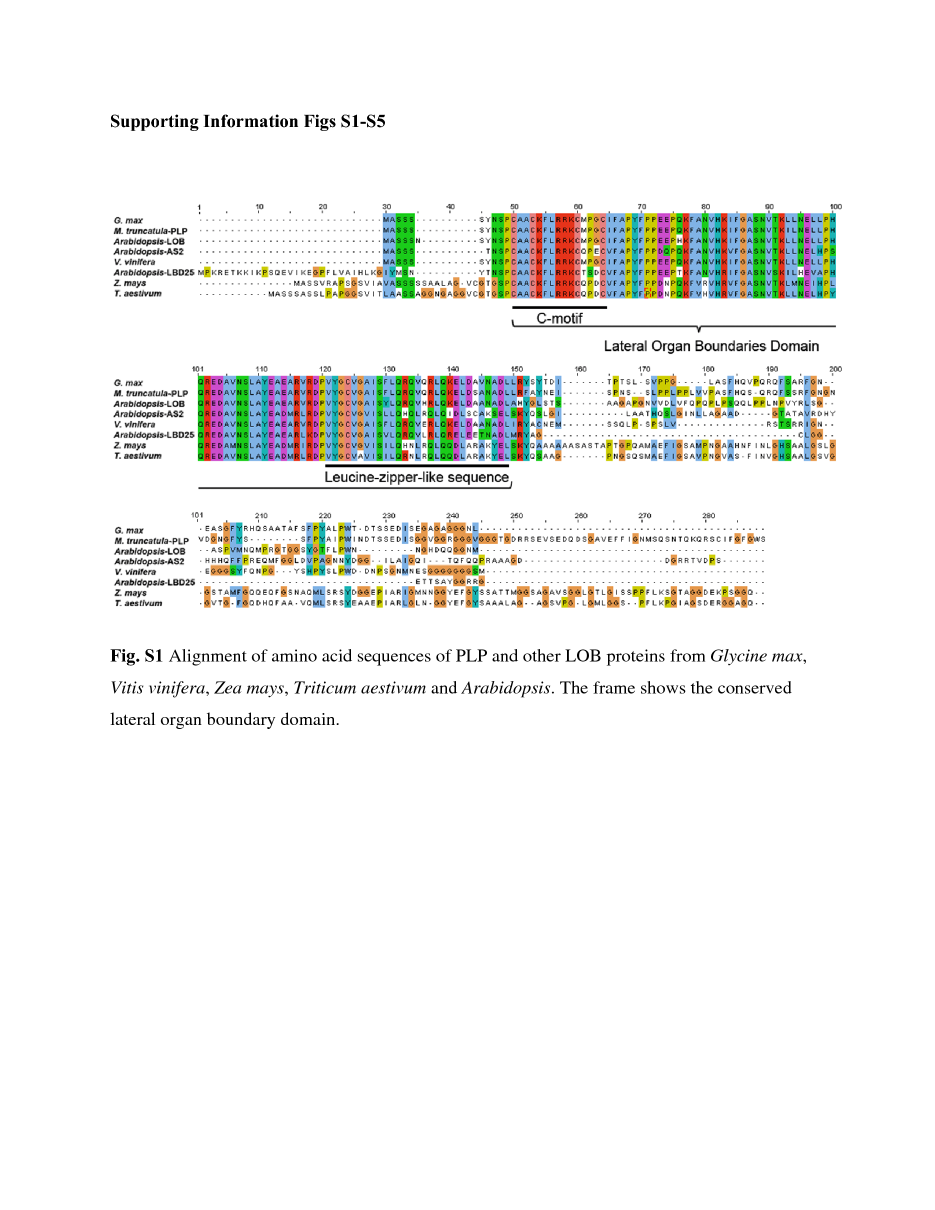

Supplement: Supplementary file 2 [file nph0196-0092-SD6.png]
